# Projected Health and Economic Impact of Long-Term Lifestyle Interventions among Older Adults

**DOI:** 10.1093/geroni/igaf122.2335

**Published:** 2025-12-31

**Authors:** Xueying Guo, Cynthia Chen

**Affiliations:** National University of Singapore, Singapore, Singapore; National University of Singapore, Singapore, Singapore

## Abstract

Singapore’s rapidly aging population presents an urgent challenge for healthcare sustainability. As chronic disease prevalence rises, proactive policy interventions are essential to mitigate future healthcare costs and disease burdens. Using the Future Elderly Model, a dynamic Markov microsimulation, we projected chronic disease trends and hospitalization costs from 2020 to 2050 while evaluating four HealthierSG-inspired interventions: (i) improved blood pressure management, (ii) increased physical activity, (iii) sodium reduction, and (iv) all combined. Without intervention, diabetes, heart disease, hypertension, stroke, obesity, and disability will increase, placing greater strain on Singapore’s healthcare system. Lifetime hospitalization costs were projected to be highest for Indians (US$93,900), followed by Chinese (US$75,700) and Malays (US$70,000). Although Malays had a higher chronic disease burden, their lower lifetime costs were due to shorter life expectancy. Implementing interventions could save up to US$505 million, with the Chinese benefiting the most, followed by Malays and Indians. These savings highlight the economic value of long-term preventive measures. This study underscores the critical role of preventive care in reshaping Singapore’s healthcare landscape. Investing in sustainable lifestyle interventions can curb rising disease rates, reduce financial strain, and improve long-term health outcomes. Policymakers must prioritize prevention strategies tailored to diverse population needs, ensuring a healthier and more sustainable future for Singapore.

